# Very high-protein and low-carbohydrate enteral nutrition formula and plasma glucose control in adults with type 2 diabetes mellitus: a randomized crossover trial

**DOI:** 10.1038/s41387-018-0053-x

**Published:** 2018-08-30

**Authors:** Maureen B. Huhmann, Shinobu Yamamoto, Joel M. Neutel, Sarah S. Cohen, Juan B. Ochoa Gautier

**Affiliations:** 1 Clinical Sciences, Nestlé Health Science, 1007 US Highway 202/206, Building JR2, Bridgewater, NJ 08807 USA; 2 Orange County Research Center, 14351 Myford Road, Suite B, Tustin, CA 92780 USA; 3 EpidStat Institute, 2100 Commonwealth Blvd, Suite 203, Ann Arbor, MI 48105 USA; 4 Department of Critical Care Medicine, Geisinger Medical Center, Danville, PA 17822 USA; 5 Department of Surgery, University of Pittsburgh, Pittsburgh, PA 15213 USA

## Abstract

**Background and objectives:**

Standard enteral nutrition (EN) formulas can  worsen hyperglycemia in diabetic patients. We hypothesized that altering the proportion of macronutrients in a formula; increasing protein while decreasing carbohydrate concentrations would improve glycemic response. The objective of this study was to demonstrate that an EN formula containing a very high concentration of protein (in the form of whey peptides) and low concentration of carbohydrate provide better control of postprandial blood glucose relative to a very high-protein/higher-carbohydrate formula.

**Subjects and methods:**

This was a randomized crossover clinical trial of 12 ambulatory adult subjects with type 2 diabetes. The primary outcome was glycemic response following a bolus of isocaloric amounts of two EN formulas; the secondary outcome was insulin response. Subjects were randomized to the experimental or the control formula, on two separate days, 5–7 days apart.

**Results:**

Mean blood glucose concentrations at 10–180 min post-infusion and mean area under the curve for glucose over 240 min post-infusion were significantly lower with the experimental formula than with the control formula (71.99 ± 595.18 and 452.62 ± 351.38, respectively; *p* = 0.025). There were no significant differences in the mean insulin concentrations over time, insulinogenic indices, and first-phase insulin measurements.

**Conclusions:**

An EN formula containing high-protein and low-carbohydrate loads can significantly improve glucose control in subjects with type 2 diabetes in ambulatory settings as evidenced by observed improved glucose control without significant difference in insulin response.

## Introduction

The use of home enteral nutrition (EN) in the United States has increased dramatically since early 1990s, with 139 per 100,000 individuals (of which 56.8% were adults) estimated to have used home EN in 2013^1^. According to Center for Disease Control and Prevention, an estimated 30.3 million individuals (9.4% of the people at least 18 years old) are living with diagnosed or undiagnosed diabetes^2^. Therefore, it would be reasonable to expect that at least 9.4% of home enterally fed patients also are diabetic. Diet is central to the management of blood glucose levels in patients with diabetes, particularly those with type 2 diabetes. Traditionally, the dietary management of the patient with diabetes has focused on decreasing carbohydrate loads and/or using carbohydrates with lower glycemic index. Significant evidence of the benefit of protein and certain lipids in the control of blood glucose is observed in volunteers and in ambulatory patients. In particular, the use of whey protein and peptides demonstrated a significant improvement in blood glucose regulation in response to carbohydrate challenge^3,4^. Although the finding needs to be confirmed, a potential beneficial effect on insulin-mediated glucose metabolism has also been observed with the use of medium chain triglycerides (MCTs) in a small study^5^. The mechanisms of how whey protein and MCTs help regulate blood sugar are incompletely understood, but may involve an increase in the release of insulin, and possibly an increase in insulin sensitivity^4,5^. Similarly, a number of studies suggest that delivering fewer calories from carbohydrates is well tolerated without a significant negative effect on clinical outcomes^6–9^.

EN formulae high in protein are prescribed to patients with a variety of conditions and diseases including protein malnutrition, muscle wasting, surgical and non-healing wounds, and critical illness. Protein needs increase with acute and chronic illnesses as well as with age^10,11^. In addition, as the caloric provision from protein in an enteral formula increases, the caloric provision from carbohydrate can decrease, which may benefit patients with type 2 diabetes.

Based on the observations above, we hypothesized that a nutritionally complete EN formula containing very high amounts of whey protein, MCTs, and a low-carbohydrate concentration could potentially improve glucose control above that of other high protein enteral nutrition formulae. We examined the effect of two different EN formulas on glucose control and insulin release.

## Materials/subjects and methods

A randomized crossover clinical trial of 12 adults with type 2 diabetes was designed to measure the primary outcome of glycemic responses and the secondary outcome of insulin response following a bolus ingestion of an isocaloric amount of two EN formulas. During the first visit, subjects were randomized to receive 450 ml of either the experimental formula containing 37% calories from hydrolyzed whey protein and 29% of calories from carbohydrates (Peptamen Intense VHP, Nestlé Health Science, Bridgewater, NJ)^12^ or the control formula containing 35% calories from whey and casein peptides along with 45% of calories from carbohydrates (Vital High Protein, Abbot Nutrition, Columbus OH)^13^ (Table 1). The randomization was performed using a Williams design appropriate for the 2 × 2 nature of this crossover trial (2 products and 2 time periods)^14^ by a statistician. A random number generator was used for sequence generation. Subjects, but not the investigators, were blinded as to the product assignments. After a 5–7 day washout period, subjects were crossed over to receive the alternate formula. Both formulas were in liquid form, kept at room temperature prior to consumption and were delivered through a nasogastric tube over 30 min.

**Table 1:** Description of the macronutrient profiles for the two test formulas

Formulas (per 450 ml)		Experimental	Control
Calories	kcal	450	450
Total protein	Amount (g)	42	39
	Source	Enzymatically hydrolyzed whey	Whey protein hydrolysate, partially hydrolyzed sodium caseinate
Total carbohydrate	Amount (g)	34	51
	Source	Maltodextrin, corn starch	Corn maltodextrin, sugar, cellulose gel
Total fat	Amount (g)	17	10
	Source	Medium chain triglycerides, fish oil, high linoleic safflower oil, soybean oil	Medium chain triglycerides, marine oil, corn oil
Dietary fiber	Amount (g)	2	0
	Source	Fructooligosaccharide, inulin	

The study recruitment and data collection took place between August and September, 2016, at Orange County Research Center, Tustin, CA, USA. Subjects were recruited from the research facility database, and all subjects had a diagnosis of type 2 diabetes well controlled with diet or diet and an oral agent. Subjects using long-acting hypoglycemic agents such as sulfonylureas, meglitinides, and alpha glucosidases inhibitors were excluded (Supplemental Table S1) to avoid potential for hypoglycemia. Other inclusion criteria were age 20–75 years old, a Hemoglobin A1C (HbA1C) less than 9.0%, and fasting glucose less than 9.99 mmol/l. Subjects were excluded if they met any of the following criteria; abnormal thyroid function, creatinine > 176.80 µmol/l, potassium < 3.5 mmol/l, gastrointestinal disease (ulcer, gastritis, diarrhea, gastroparesis, and vomiting), history of gastric bypass surgery, midface trauma, esophageal varices, and coagulation abnormalities, on any anti-coagulant medication, unstable diabetes, under treatment for cancer, heart disease, or renal disease, unable to give informed consent or follow instructions, insulin therapy, pregnant, allergies to milk, fish oil, or any component of the test product, and participating in another competing clinical trial.

### Ethics

This trial was approved (IRB tracking #: NES1-16-370) by Copernicus Group IRB, Durham, NC, USA, and fulfilled all requirements for human research, including Declaration of Helsinki and Good Clinical Practice. The trial was posted on clinicaltrials.gov (ClinicalTrials.gov Identifier: NCT02898766).

### Clinic visits

Each patient had a screening visit and two subsequent test visits separated by a 5–7 day washout period. Informed consent, medical history a brief physical examination, and samples for serum chemistry (glucose serum, sodium, blood urea nitrogen, calcium, chloride, HbA1C, creatinine, potassium, thyroid stimulating hormone, T-4) were obtained at the screening visit. Subjects were instructed to fast after midnight. Following a weight check, an intravenous line for blood withdrawal was placed, and baseline (0 min) blood samples for fasting glucose and insulin concentrations were collected. Subjects were instructed not to consume oral diabetes medications, smoke, or consume any non-study related foods or beverages before or during the 4-h test visits. Following placement of a nasogastric tube by the study staff, the test formula was infused over 30 min using a 60 ml syringe, following which the tube was immediately removed. Blood samples for glucose and insulin concentrations were drawn at 10, 20, 30, 60, 90, 120, 150, 180, 210, and 240 min following formula consumption. Unintended effects of the interventions were assessed at each visit. No adverse events occurred during the course of the study.

### Laboratory measurements

At each test visit, up to a total of 80 ml of serum/plasma were obtained from blood samples through an indwelling catheter, drawn by one of the research team staff. Glucose was analyzed by UV test using Roche modular, and insulin was analyzed by solid-phase, two-site chemiluminescent immunometric assay using IMMULITE 2000, at Consolidated Medical Bio-Analysis, Cypress, CA, USA.

### Sample size

In a previous study, the standard deviation of the difference in area under the curve (AUC) for blood glucose concentration between two different EN formulas was 4.11 mmol/l/4 h^15^. In this study, the sample size of 11 subjects were required to detect a difference of 5.00 mmol/l/4 h between two EN formulas with type I error rate of 0.05 and power of 0.8. Twelve subjects were enrolled and all of them completed the study.

### Statistical analyses

The paper-based case report forms were entered into an Excel database and checked for errors by a statistician. Electronic laboratory data were linked to the database using patient ID. Statistical analyses were performed using SAS/STAT software, version 9.3 (SAS Institute Inc., Cary, NC). The data met statistical assumptions. Unless otherwise stated, *p*-value < 0.05 was used to determine statistical significance, and all p-values reported were two-sided. The lower and upper detection limits for insulin test were 2 and 300 µIU/ml, respectively. Therefore, to avoid extrapolation, when insulin values were <2 or >300 µIU/ml, the values 2 and 300 µIU/ml, respectively, were used in the analyses. AUC (mmol/l/4 h), which represents a response over the course of the study, was calculated using the trapezoid rule for each patient for blood glucose concentration, and difference in the means of AUC between the formulas were assessed by a random effects model^16^. The difference in the mean glucose or mean insulin concentrations between formulas were assessed using a crossover t-test at each time point. The difference in mean glucose at each time point from the corresponding baselines within formula was assessed using a modified alpha-level with the Bonferroni correction. For each patient, peak blood glucose concentration (*C*_max_) (mmol/l), time of *C*_max_ (*T*_max_) (min), and first-phase insulin secretion AUC (µIU/ml/0.5 h) from 0 to 30 min were calculated, in addition to, insulinogenic index, $\left( {\frac{{\Delta insulin}}{{\Delta glucose}}} \right)^\ast 0.0555$ (µIU/mmol), where Δinsulin and Δglucose denote the difference in insulin and glucose concentrations at baseline and 30 min post-infusion, respectively, and 0.0555 was used to convert glucose from mg/dl to mmol/l^17^. The difference in the first-phase insulin secretion or the mean insulinogenic indices between the formulas were assessed using crossover t-tests.

## Results

Out of 17 subjects screened, 5 were screen failures, and 12 were randomized into the trial. Twelve subjects completed the trial and were included in the analyses. No adverse events occurred during the course of the study. The subjects had mean age of 56 ± 7.5 years (range 20–75 years) and 50% were female. There were six Caucasians, three African Americans, two Hispanics, and one subject reported other race. The mean BMI was 33.5 ± 5.5, and ten (83%), eight (67%), and nine (75%) subjects had hypertension, hyperlipidemia, and were receiving metformin, respectively (Table 2).

**Table 2:** Patients characteristics at the trial enrollment (*N* = 12)

Characteristic		*N* (%) or Mean ± SD
Race	Caucasian	6 (50%)
	African American	3 (25%)
	Hispanic	2 (17%)
	Other	1 (8%)
Sex	Female	6 (50%)
	Male	6 (50%)
Age (years)		56.0 ± 7.5
Height (cm)		172.3 ± 12.8
Weight (kg)		99.5 ± 19.0
Body mass index (kg/m^2^)		33.5 ± 5.5
Hemoglobin A1C (%)^a^		6.8 ± 1.2
Systolic blood pressure (mmHg)		126.5 ± 12.9
Diastolic blood pressure (mmHg)		79.9 ± 5.9
Heart rate (bpm)		76.8 ± 8.0
Comorbidities	Hypertension	10 (83.3%)
	Hyperlipidemia	8 (66.7%)
	Neuropathy	1 (8.3%)
Medication usage	Metformin	9 (75.0%)
	Dietary management alone	3 (25.0%)
	Antihyperlipidemic drugs	4 (33.3%)
	Antihypertensive drugs	8 (66.7%)
Other drugs (i.e., analgesic, clotting prophylaxis, thyroid replacement)		7 (58.3%)

*SD* standard deviation

^a^geometric mean ± SD

### Blood glucose response to the formulas

**Fig. 1:**
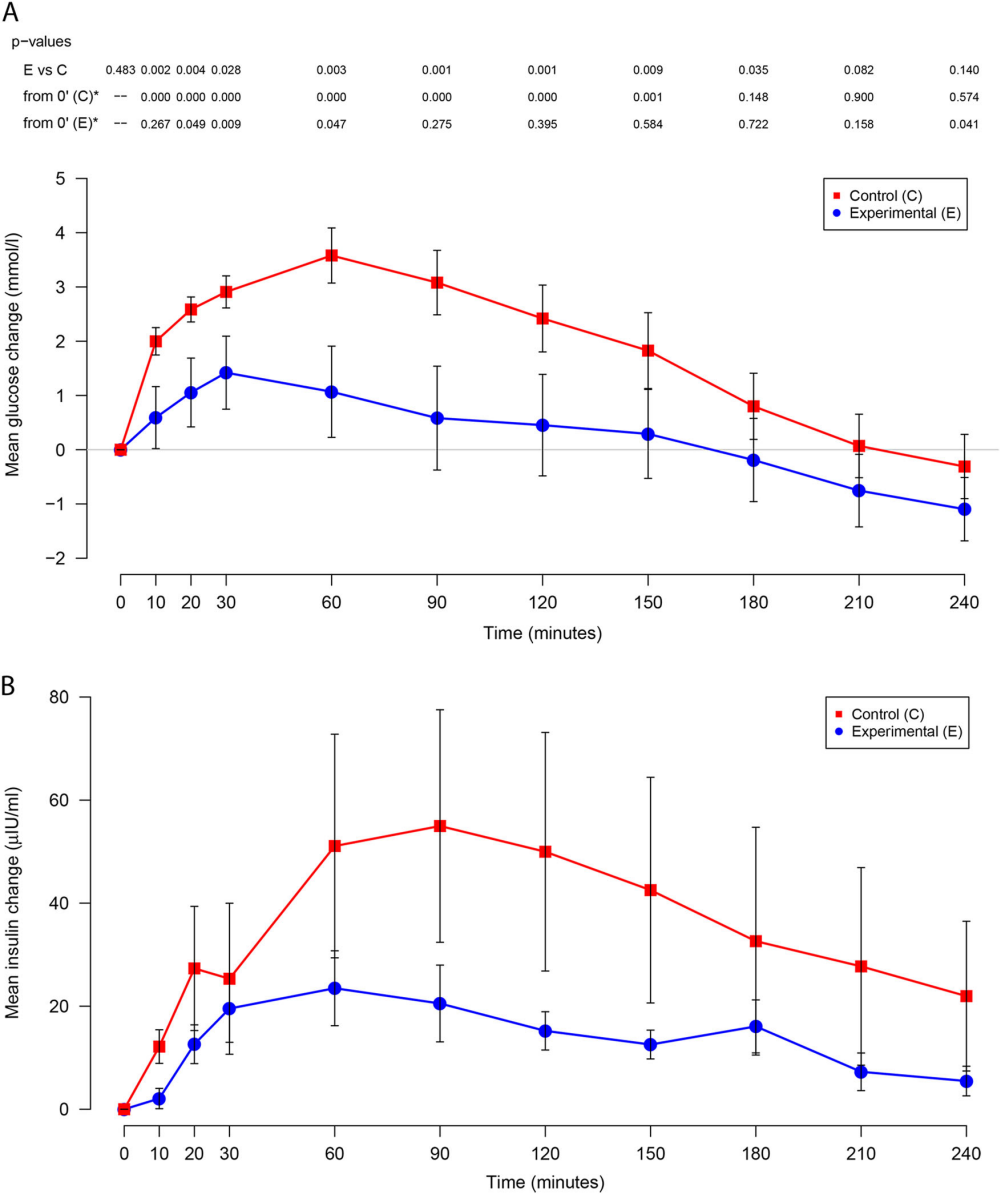
Change in blood glucose and insulin concentrations from baseline over time among patients receiving experimental and control formula (*N* = 12). Change in **a** blood glucose (mmol/l) and **b** insulin (µIU/ml) concentrations from baseline over time (minutes) post infusion by formula are shown. The experimental (**E**) formula is shown in blue, and the control (**C**) formula is shown in red. Bars represent standard error of the mean. The p-values for testing if blood glucose concentration was different from baseline at each time point within formulas (from 0’ (**C**)* and from 0’ (**E**)*, *: statistical significances were observed at a modified alpha-level of 0.005 with the Bonferroni correction) and for testing if blood glucose concentrations were different between the two formulas (**E** vs **C**) at each time point are shown on the top of the plot

The change in glucose concentrations from baseline over 240 min for each formula are illustrated in Fig. 1a. The mean blood glucose concentrations for the experimental and the control formulas were 7.58 ± 2.09 and 7.20 ± 1.66 mmol/l, respectively, and were comparable at baseline (*p* = 0.48). Compared to the baseline (0 min), there was a significant increase in blood glucose concentrations within 10 min in response to a dietary challenge with the control formula (adjusted *p* < 0.005), which lasted until 150 min. In contrast, only moderate increases in blood glucose concentrations from baseline occurred at 30 min with experimental formula (adjusted *p* = 0.009), with no further increase in blood glucose across time. Between the formulas, the increase in blood glucose was significantly lower when the subjects were given the experimental formula compared to the control formula across eight time points from 10 to 180 min (*p* < 0.05). Blood glucose returned to the baseline level at 180 min with control formula (adjusted *p* > 0.1), and were no longer significantly different between the two formulas at 210 and 240 min (*p* > 0.05). The minimum and maximum of the mean glucose concentrations (mmol/l) for the experimental formula were smaller than those of the control formulas over the course of the trial [(6.48 ± 2.41, 9.00 ± 1.71) and (6.90 ± 2.79, 10.78 ± 2.47), respectively].

The AUCs for glucose are shown for each subject in Table 3. The mean AUC for glucose for the experimental formula was smaller than that of the control formula (71.99 ± 595.18 and 452.62 ± 351.38, respectively; *p* = 0.03). Individual AUCs were smaller in 11 of 12 subjects receiving the experimental rather than for the control formula (Table 3, AUC: Control—AUC: Experimental) as expected.

**Table 3:** Area under the curve (AUC), Maximum value (*C*_max_), and time of maximum value (*T*_max_) for glucose values in individual patient receiving experimental formula or control formula (*N* = 12)

Patient	AUC: Experimental	AUC: Control	*C*_max_: Experimental	*C*_max_: Control	*T*_max_: Experimental	*T*_max_ :Control
101	538.9	862.47	13.76	14.04	120	90
102	135.7	319.12	6.55	7.33	30	10
103	988.73	1234.6	12.76	14.6	150	150
104	−1514.32	254.47	7.05	10.49	30	30
105	228.1	224.5	8.82	11.16	60	60
106	−57.72	159.56	8.27	9.77	60	60
107	−191.47	193.42	9.27	12.04	120	60
108	308.02	666.55	9.27	11.65	90	60
109	169.28	778.66	8.38	12.54	30	90
110	302.47	376.57	10.66	11.65	20	150
111	−210.62	107.11	10.21	11.38	30	60
112	166.78	254.47	7.99	9.05	60	30
Mean	71.99	452.62			66.7	70.8
SD	595.18	351.38			43.6	43.6
*p*-value	*p* = 0.025				*p* = 0.780	

Peak blood glucose concentration (*C*_max_) and time of *C*_max_ (*T*_max_) are also shown in Table 3. The peak glucose concentrations of the experimental formula were consistently lower than those of the control formula for all subjects (Table 3, *C*_max_: Control—*C*_max_: Experimental). The time to achieve the highest glucose value (*T*_max_) varied between subjects with some achieving their highest values within 10 min and others up to 150 min. Interestingly, the formulas also influenced the *T*_max_ with some subjects achieving *T*_max_ that were widely different between formulas. Overall, *T*_max_ for the experimental group occurred at 66.7 ± 43.6 min as compared to 70.8 ± 43.6 min (*p* = 0.78) in the control group.

### Endogenous insulin production

The change in insulin concentration from baseline over 240 min by formulas are shown in Fig. 1b and are characterized by significant variations between individuals. The mean insulin concentrations were not statistically different at baseline between the formulas (11.4 ± 7.2 and 9.2 ± 6.8 µIU/ml for experimental and control, respectively; *p* = 0.23). There was a trend towards lower average endogenous insulin production in response to the experimental group over time (10–240 min) when compared to the control group (*p* > 0.1). The minimum and maximum of the mean insulin (µIU/ml) for the experimental and the control formulas were (11.4 ± 7.2, 34.9 ± 26.0) and (9.2 ± 6.8, 64.2 ± 81.6), respectively. The mean insulinogenic indices for the experimental and the control formulas were 10.9 ± 12 and 6.6 ± 10.4, and the mean first-phase insulin measures (AUC 0–30 min) were 244.6 ± 227.6 and 521.5 ± 749.3, respectively. There were no significant difference between the formulas for these outcomes (*p* = 0.15; *p* = 0.23, respectively; data not shown).

## Discussion

This study demonstrates that the differences in macronutrient composition, specifically carbohydrate, protein, and fat, in EN formula can exert a significant metabolic effect on blood glucose levels of subjects with type 2 diabetes. The experimental and the control formulas tested in the current study contain very high amounts of protein, providing 37 and 35% of total calories, respectively (Table 1). The improved glycemic response observed in this study is most likely due to a reduction in glycemic load, whereby carbohydrate is replaced by increasing protein and fat concentrations. However, there may be an additional explanations for the significant difference in glucose control. In particular, the type of protein in the two formulas varies. The experimental formula contains hydrolyzed protein sourced from 100% whey. Whey has been associated with an increased release of insulin and lower blood sugar levels^3,18,19^. There are several potential mechanisms for this proposed possible insulinotropic effect of whey. The higher leucine content appears to stimulate insulin secretion through the mammalian target of rapamycin pathway^20^. Whey stimulates an increase in incretin hormone glucagon-like protein-1 (GLP-1)^21^ which in turn may delay gastric emptying^22^. Additionally, inhibition of dipeptidyl peptidase-IV by whey derived bioactive peptides^23^ leads to increased half-lives of GLP-1 and glucose-dependent insulinotropic polypeptide. There is also a potential effect of MCT on glycemic control^5^. Furthermore, fish oil may also play a key role in increasing adiponectin resulting in improved insulin sensitivity^24^. The experimental formula did contain a very small amount of fiber, however the presence or absence of two grams of fiber in the formulas was an unlikely cause of the differential glucose responses as consumption of larger amount of fructooligosaccharides for extended periods did not affect fasting blood glucose in patients with type 2 diabetes as shown by Alles^25^.

While a statistically significant difference in insulin responses was not observed between the experimental and the control formulas, a trend towards a lower insulin release was observed with the experimental formula. There are a few possible explanations for this observation. First, the experimental formula contained a smaller amount of carbohydrate compared to the control formula, resulting in a lower blood glucose and lower insulin response. Second, the large variances in the insulin response with the control formula may have caused the lack of statistical difference. It should be noted that the study was not powered to detect the significant difference in insulin response by design. Finally, the experimental formula possibly induced insulin secretion above what was expected by the amount of carbohydrate in the formula alone; for example, additional induction of insulin release by other ingredients such as whey, MCT, and fish oil as mentioned above. Additional experiment is needed to support this theory.

The low-carbohydrate diet has been demonstrated to improve glucose management in patients with type 2 diabetes^26^. However, diabetic enteral formulas typically have lower protein concentrations and tend to have a higher proportion (40–50% of total calories) in lipids^27^ and therefore may not be adequate for patients with increased protein requirements. For example, skeletal muscle wasting and function loss is observed in acutely and chronically ill individuals^28,29^, but essential amino acid supplementation during the inactivity appears to assist with preservation of muscle function^30^. Additionally, it has been observed that increased protein intake assists in the retention of lean body mass in the elderly^31,32^. The source of protein seems to affect its ability to influence muscle. For example, whey contains a higher concentration of leucine which is associated with muscle protein synthesis in the elderly when compared to casein or hydrolyzed casein^33^. Furthermore, there is a positive association between protein intake and bone density in elderly populations although additional studies are needed to validate the findings^34,35^. Therefore, EN formulas with a higher proportion and quality of protein may better meet the nutritional requirements of these patients.

The crossover design, in which glucose and insulin responses to the two formulas were examined within same subjects, strengthens this study. The results of this study are limited by the fact that the subjects received a single bolus of 450 ml of formula. Although this design allowed us to observe the acute effect of the two formulas on glucose and insulin concentrations and is directly applicable to patients receiving bolus EN, it may not produce as dramatic results in patients receiving continuous EN.

In conclusion, this study demonstrates that use of a very high protein, low carbohydrate formula is associated with a reduced glycemic response in subjects with type 2 diabetes. The formula may assist in improving blood glucose management in long term enterally fed subjects with type 2 diabetes. It is possible that the formula may also benefit other patients who require better blood glucose control that also have increased protein needs.

## Electronic supplementary material

Supplementary Table S1

## References

[1] Mundi MS, Pattinson A, McMahon MT, Davidson J, Hurt RT (2017). Prevalence of home parenteral and enteral nutrition in the United States. Nutr. Clin. Pract..

[2] Centers for Disease Control and Prevention. *National Diabetes Statistics Report, 2017* (Atlanta, GA: Centers for Disease Control and Prevention, US Department of Health and Human Services, 2017).

[3] Acheson KJ (2011). Protein choices targeting thermogenesis and metabolism. Am. J. Clin. Nutr..

[4] Adams RL, Broughton KS (2016). Insulinotropic effects of whey: mechanisms of action, recent clinical trials, and clinical applications. Ann. Nutr. Metab..

[5] Eckel RH (1992). Dietary substitution of medium-chain triglycerides improves insulin-mediated glucose metabolism in NIDDM subjects. Diabetes.

[6] Ceriello A, Lansink M, Rouws CH, van Laere KM, Frost GS (2009). Administration of a new diabetes-specific enteral formula results in an improved 24h glucose profile in type 2 diabetic patients. Diabetes Res. Clin. Pract..

[7] Han, Y. Y. *et al*. The clinical and economic impact of the use of diabetes-specific enteral formula on ICU patients with type 2 diabetes. *Clin Nutr.* **36**;(6):1567–1572. 10.1016/j.clnu.2016.09.027. Epub 2016 Oct 6. PubMed PMID: 27765525. 10.1016/j.clnu.2016.09.027 27765525

[8] Lansink, M., Hofman, Z., Genovese, S., Rouws, C. H., Ceriello, A. Improved glucose profile in patients with type 2 diabetes with a new, high-protein, diabetes-specific tube feed during 4 h of continuous feeding. *J*. *Parenter*. *Enteral*. *Nutr*. (2016). 10.1177/0148607115625635 26826263

[9] Mesejo A (2015). Diabetes-specific enteral nutrition formula in hyperglycemic, mechanically ventilated, critically ill patients: a prospective, open-label, blind-randomized, multicenter study. Crit. Care..

[10] Bauer J (2013). Evidence-based recommendations for optimal dietary protein intake in older people: a position paper from the PROT-AGE Study Group. J. Am. Med. Dir. Assoc..

[11] Paddon-Jones D, Short KR, Campbell WW, Volpi E, Wolfe RR (2008). Role of dietary protein in the sarcopenia of aging. Am. J. Clin. Nutr..

[12] PEPTAMEN® INTENSE HIGH PROTEIN. https://www.nestlehealthscience.ca/en/brands/peptamen/peptamen-intense-hp. Accessed 18 January 2018.

[13] Vital® High Protein. https://abbottnutrition.com/vital-high-protein. Accessed 18 January 2018.

[14] Wang BS, Wang XJ, Gong LK (2009). The construction of a williams design and randomization in cross-over clinical trials using SAS. J. Stat. Softw..

[15] Hofman, Z. R. C., van Drunen, J., De Later, C., Kuipers, H. (eds.) The effect of enteral nutrition on glucose and triglyceride concentrations during 6h continuous feeding in diabetic patients. In *26th ESPEN (European Society Clinical Nutrition and Metabolism) Congress, 2004*. (Lisbon, Portugal, 2004).

[16] Yarandi, H. N. Paper SD04: crossover designs and proc mixed in SAS. *SouthEast SAS Users Group Meeting* (2004).

[17] Seltzer HS, Allen EW, Herron AL, Brennan MT (1967). Insulin secretion in response to glycemic stimulus: relation of delayed initial release to carbohydrate intolerance in mild diabetes mellitus. J. Clin. Invest..

[18] Akhavan T, Luhovyy BL, Brown PH, Cho CE, Anderson GH (2010). Effect of premeal consumption of whey protein and its hydrolysate on food intake and postmeal glycemia and insulin responses in young adults. Am. J. Clin. Nutr..

[19] Morifuji M (2010). Comparison of different sources and degrees of hydrolysis of dietary protein: effect on plasma amino acids, dipeptides, and insulin responses in human subjects. J. Agric. Food Chem..

[20] Yang J (2012). Leucine stimulates insulin secretion via down-regulation of surface expression of adrenergic alpha2A receptor through the mTOR (mammalian target of rapamycin) pathway: implication in new-onset diabetes in renal transplantation. J. Biol. Chem..

[21] Luscombe-Marsh ND, Hutchison AT, Soenen S, *et al*. Plasma Free Amino Acid Responses to Intraduodenal Whey Protein, and Relationships with Insulin, Glucagon-Like Peptide-1 and Energy Intake in Lean Healthy Men. Nutrients. **8**(1):4. 10.3390/nu8010004 (2016). 10.3390/nu8010004 PMC4728618 26742062

[22] Deane AM (2010). Endogenous glucagon-like peptide-1 slows gastric emptying in healthy subjects, attenuating postprandial glycemia. J. Clin. Endocrinol. Metab..

[23] Lacroix IM, Li-Chan EC (2014). Isolation and characterization of peptides with dipeptidyl peptidase-IV inhibitory activity from pepsin-treated bovine whey proteins. Peptides.

[24] Wu JH, Cahill LE, Mozaffarian D (2013). Effect of fish oil on circulating adiponectin: a systematic review and meta-analysis of randomized controlled trials. J. Clin. Endocrinol. Metab..

[25] Alles MS (1999). Consumption of fructooligosaccharides does not favorably affect blood glucose and serum lipid concentrations in patients with type 2 diabetes. Am. J. Clin. Nutr..

[26] Gannon MC, Nuttall FQ (2004). Effect of a high-protein, low-carbohydrate diet on blood glucose control in people with type 2 diabetes. Diabetes.

[27] Davidson P, Kwiatkowski CA, Wien M (2015). Management of hyperglycemia and enteral nutrition in the hospitalized patient. Nutr. Clin. Pract..

[28] Gruther W (2008). Muscle wasting in intensive care patients: ultrasound observation of the M. quadriceps femoris muscle layer. J. Rehabil. Med..

[29] Lieffers JR, Bathe OF, Fassbender K, Winget M, Baracos VE (2012). Sarcopenia is associated with postoperative infection and delayed recovery from colorectal cancer resection surgery. Br. J. Cancer.

[30] Ferrando AA (2010). EAA supplementation to increase nitrogen intake improves muscle function during bed rest in the elderly. Clin. Nutr..

[31] Houston DK (2008). Dietary protein intake is associated with lean mass change in older, community-dwelling adults: the Health, Aging, and Body Composition (Health ABC) Study. Am. J. Clin. Nutr..

[32] Kerstetter JE (2015). The effect of a whey protein supplement on bone mass in older caucasian adults. J. Clin. Endocrinol. Metab..

[33] Pennings B (2011). Whey protein stimulates postprandial muscle protein accretion more effectively than do casein and casein hydrolysate in older men. Am. J. Clin. Nutr..

[34] Promislow JH, Goodman-Gruen D, Slymen DJ, Barrett-Connor E (2002). Protein consumption and bone mineral density in the elderly: the Rancho Bernardo Study. Am. J. Epidemiol..

[35] Schurch MA (1998). Protein supplements increase serum insulin-like growth factor-I levels and attenuate proximal femur bone loss in patients with recent hip fracture. A randomized, double-blind, placebo-controlled trial. Ann. Intern. Med..

