# Supplementary material for: Very high-protein and low-carbohydrate enteral nutrition formula and plasma glucose control in adults with type 2 diabetes mellitus: a randomized crossover trial
Source: Nutr Diabetes. 2018 Aug 30;8:45. doi: 10.1038/s41387-018-0053-x (PMC6115411; doi:10.1038/s41387-018-0053-x)
Supplement: Supplementary file 1 — Supplementary Table S1 [file 41387_2018_53_MOESM1_ESM.docx]

**Supplemental Table S1:** **Exclusion criteria - antihyperglycemic drugs**

| Drug class | Generic name (example) |
| --- | --- |
| Sulfonylureas | Glimepiride |
|  | Glipizide |
|  | Glyburide |
| Meglitinides | Repaglinide |
|  | Nateglinide |
| Alpha-glucosidase inhibitors | Miglitol |
|  | Acarbose |
